# The Graham Journal of Health and Longevity

**Published:** 1837

**Authors:** 


Art. VI.—The Graham Journal of Health and Longevity.

Such is the title of a hebdominal of eight octavo pages, issued weekly from a Boston press. Its object is to put an end to the use of animal food, which is placed on nearly the same footing with the use of ardent spirits! The leading argument is, that apes and monkeys are frugivorous animals—the digestive organs of man bear a close resemblance to theirs; *ergo*, he should live on the same diet. We commend to the Graham school another syllogism:—The hands and feet of man resemble those of the baboon more than those of any other animal; but the baboon lodges among the green branches; therefore, man should abandon his present domitory, for the limbs of a tree. Again: our teeth and jaws resemble those of the ape; but the ape does not drink milk; *ergo*, we should not—and yet it makes an important item in the Graham bill of fare! That many persons in the United States, where meat is so abundant, devour a much larger quantity than is salutary, we have no doubt; but those who cannot see in this excessive indulgence, the proof of a natural taste for flesh, give but sorry evidence of the boasted influence of a vegetable diet on the intellectual faculties. Every mother, in every country where animal food is attainable, knows that children *prefer* it to vegetable. Indeed, it would appear to be their proper diet, for Nature supplies them with it from the beginning; and where God forms the desires, the necessities and the supplies are always in harmony. Why does not Mr. Graham prohibit his followers from the use of milk, as well as cheese and butter? In practice, he violates his own dogmas. Why does he allow them wheat bread, the gluten of which has nearly the composition of muscle? Why does he prohibit eggs, to which his standard, the monkey race, are so addicted? We advise the Graham school to look a little closer into this matter. They will find that an exclusively vegetable diet, is quite a chimera. The herbivorous animals take in no small quantity of animal food. The water they drink very commonly abounds in animalcules; and the grass and herbage on which they feed, swarm with insects and worms. If animal food is not natural to man, and proper for his health, why did he use it in the very infancy of society? What could prompt him but his appetite? Mr. Graham and his disciples are Christians. How then do they dispose of the third verse of the ninth chapter of Genesis, and of every subsequent recognition of the same kind, displayed on the pages of the Old and New Testaments? We have no personal objection to their abstinence. We are not ourselves *carnivora* in practice, and, indeed, take but little meat. But we believe that man was formed for a mixed diet, and will continue to eat it. We know that many persons are injured by an excessive proportion of vegetable food—others by too much animal. We are convinced that the best moral, intellectual and physical developments of our race, have been made under a mixed diet;—beginning with the Hebrew patriarchs, and descending to the nations of modern Europe, and the States of this Union. We believe, that the universal adoption of the grano-fructo-herbivorous system, would speedily deteriorate society; but we believe, also, that no such adoption need be apprehended. Why, then, it may be asked, indite these paragraphs against that system? We answer, because many persons (several of whom have already fallen under our observation) are likely to be injured by it. On the other hand, many, it is true, may be benefited. But, it is a bad system which produces one evil while it corrects another. Such, however, is any kind of fanaticism. Of all reformers fanatics are the most dangerous. Their vision is directed to a single point, and remains fixed on one object, till *that* object becomes *all in all.* They see a speck in the sky, and pronounce that a tempest is at hand; you point to the clear blue vault of heaven above, the sparkling stars, or the beaming sun, as proofs of the contrary, but they see only the black spot, and consistently denounce you as improvident or profane. In every age the science of medicine has been infested with these benevolent but obstinate monomaniacs. If you encounter one of them, it is at your peril. The greater the absurdity the more obstinate its defence. Already is their Journal out upon the regular profession. The 18th number contains a paragraph, which is quite *ad captandum vulgus.* This is very natural; the medical profession must be brought into discredit, before the people will disregard its maxims; and what are its maxims on the subject of diet? Why, that man was designed by his Creator to live on a mixture of animal and vegetable food; that the proportions must be different in different individuals; that in some forms, or tendencies to disease, an animal—in others, a vegetable diet, is necessary; that in the United States *too much* of both is generally taken; and that the duty of man to himself, in this matter, is, to eat no more of either, than is requisite to satisfy the wants of his constitution; but this sober, common sense is quite too bland to suit the appetite of fanaticism—it is not sufficiently stimulating. D.

